# Comment on “Long-term impact of synchronous and metachronous bladder cancer on prognosis after radical nephroureterectomy for upper urinary tract urothelial carcinoma: results from a large population-based cohort in China”

**DOI:** 10.1097/JS9.0000000000003146

**Published:** 2025-08-06

**Authors:** Xianwen Liang, Qian Liu, Xuyang Sun

**Affiliations:** a Department of Anorectal Surgery, Hainan General Hospital (Hainan Affiliated Hospital of Hainan Medical University), Haikou, Hainan, China; b Department of Gastrointestinal Surgery, Hainan General Hospital (Hainan Affiliated Hospital of Hainan Medical University), Haikou, Hainan, China; c Department of Interventional Radiology, Hainan Hospital of the General Hospital of the People’s Liberation Army of China, Haikou, Hainan, China

*Dear Editor,*

We read with great interest the article titled “Long-term impact of synchronous and metachronous bladder cancer on prognosis after radical nephroureterectomy for upper urinary tract urothelial carcinoma: Results from a large population-based cohort in China^[1]^.” This study provides valuable insights into the impact of synchronous and metachronous bladder cancer on the prognosis of upper urinary tract urothelial carcinoma (UTUC) patients, and we commend the authors for their significant contributions to this important area of research. However, we would like to address a few concerns that may impact the completeness and applicability of the findings. This correspondence was developed following the TITAN Guidelines 2025^[2]^ for the declaration and use of artificial intelligence in scholarly publishing. No AI tools were employed in the conception, design, analysis, interpretation, or writing of this work.

While the authors conducted thorough analyses on the prognostic significance of synchronous and metachronous bladder cancer, the study does not sufficiently address the potential role of key treatment factors, such as radiotherapy and immunotherapy, which have become increasingly recognized for their significant impact on survival outcomes in UTUC patients.

Radiotherapy, particularly in cases of locally advanced or unresectable UTUC, has shown promise in improving local control and survival^[3]^, especially when combined with surgery or chemotherapy. Immunotherapy, specifically PD-1/PD-L1 inhibitors, has demonstrated substantial efficacy in metastatic or recurrent UTUC^[4]^, providing a viable treatment option for patients who do not respond well to traditional therapies like chemotherapy.

Both of these treatment modalities have been shown to significantly influence overall survival and cancer-specific survival in patients with urothelial carcinoma. The exclusion of these factors from the study’s analysis may limit the ability to fully capture the complexity of clinical outcomes in UTUC patients, as treatment regimens, such as chemotherapy, radiotherapy, and immunotherapy, can potentially confound the relationship between bladder cancer and UTUC prognosis.

We recommend that the authors consider presenting these variables in the results section in future studies. If these treatment factors are balanced between the study groups, it would not only enhance the robustness of the findings but also provide a more comprehensive picture of the impact of concurrent treatment strategies on patient survival.

## Data Availability

The letter does not have any data.

## Research registration unique identifying number (UIN)

Not registered for the type of the study.
